# Perfluorohexyloctane ophthalmic solution for dry eye disease: pooled analysis of two phase 3 clinical trials

**DOI:** 10.3389/fopht.2024.1452422

**Published:** 2024-11-05

**Authors:** Ahmad M. Fahmy, Jennifer S. Harthan, David G. Evans, Jack V. Greiner, Joseph Tauber, John D. Sheppard, Sonja Krösser, Jason L. Vittitow

**Affiliations:** ^1^ Minnesota Eye Consultants, Minneapolis, MN, United States; ^2^ Illinois College of Optometry, Chicago, IL, United States; ^3^ Total Eye Care, PA, Memphis, TN, United States; ^4^ Clinical Eye Research of Boston, Winchester, MA, United States; ^5^ Tauber Eye Center, Kansas City, MO, United States; ^6^ Virginia Eye Consultants, Norfolk, VA, United States; ^7^ Novaliq GmbH, Heidelberg, Germany; ^8^ Bausch + Lomb, Bridgewater, NJ, United States

**Keywords:** dry eye disease, tear film evaporation, Meibomian gland dysfunction, perfluorohexyloctane, clinical trial, NOV03

## Abstract

**Background:**

Dry eye disease (DED) is commonly caused by excessive tear film evaporation due to Meibomian gland dysfunction (MGD). There is a need for DED treatment options that address tear evaporation and benefit patients across a broad range of demographic and disease characteristics. This study evaluated treatment effects of perfluorohexyloctane ophthalmic drop (formerly NOV03) in the pooled dataset from 2 pivotal clinical trials in patients with DED associated with MGD, both in the overall population and in patient subgroups based on sex, age, and baseline severity of eye dryness.

**Methods:**

Pooled data from 2 similarly designed, phase 3, randomized controlled trials (GOBI, MOJAVE) were analyzed. Patients aged ≥18 years with DED administered perfluorohexyloctane (n=614) or hypotonic (0.6% solution) saline control (n=603) four times daily for 8 weeks. Primary endpoints were total corneal fluorescein staining (tCFS) score (National Eye Institute scale, 0-15) and eye dryness visual analog scale (VAS) score (0-100). Efficacy was evaluated using analysis of covariance among patient subgroups (male and female, older [≥65 years] and younger [18 to <65 years], tCFS score <7 and ≥7, VAS eye dryness score <70 and ≥70, MGD score <7 and ≥7, Schirmer I test <10 mm and ≥10 mm).

**Results:**

Reductions in tCFS and VAS eye dryness scores were greater for perfluorohexyloctane versus control. In the overall patient population, least-squares mean treatment difference was −1.1 (95% CI: −1.41 to −0.79; p<0.0001) for tCFS and −9.0 (95% CI: −11.90 to −6.00; p<0.0001) for VAS eye dryness. Treatment favored perfluorohexyloctane over control in all patient subgroup analyses of tCFS and VAS eye dryness. Overall, the most common adverse event with perfluorohexyloctane was blurred vision (2.1% of patients), which was mild and transient.

**Conclusions:**

Compared with a hypotonic saline control, perfluorohexyloctane improved both the signs and symptoms of DED, including in patients with greater self-reported severity of eye dryness.

**Clinical trial registration:**

This study represents an integrated analysis of 2 previous clinical trials: GOBI (ClinicalTrials.gov, NCT04139798) and MOJAVE (ClinicalTrials.gov, NCT04567329).

## Introduction

As defined by the Dry Eye Workshop II of the Tear Film and Ocular Surface Society, dry eye disease is “a multifactorial disease of the ocular surface characterized by a loss of homeostasis of the tear film and accompanied by ocular symptoms” (1). Symptoms of dry eye disease that may be experienced by patients include dryness, burning, stinging, grittiness, ocular fatigue, and blurred vision (2, 3). Signs of dry eye disease that may be observed during clinical examination include decreased tear film volume, tear film instability, and damage to the ocular surface (2, 3). Dry eye disease is typically categorized as aqueous deficient (in which tear production is reduced), evaporative (in which tear film evaporation is excessive), or mixed (1). In the majority of patients (>85%), dry eye disease has an evaporative component (4, 5).

The tear film consists of an inner aqueous−mucin layer and an outer lipid layer, which is composed of amphiphilic polar lipids (at the interface with the aqueous−mucin layer) and nonpolar lipids (at the air−tear interface) (6, 7). The tear film lipid layer has evaporation-resistant properties, and deficiencies in the lipid layer cause increased evaporation and thinning of the tear film (7). Excessive evaporation leads to a cascade of other effects, including tear film hyperosmolarity, desiccation stress, and ocular surface inflammation and damage, which results in the clinical signs and symptoms of dry eye disease (7, 8). Secretion from the Meibomian glands (ie, meibum) is the primary source of tear film lipids (9, 10), and Meibomian gland dysfunction has been identified as the primary cause of evaporative dry eye disease (1, 11, 12).

The management of dry eye disease often follows a stepwise approach, beginning with home-based and over-the-counter therapies (eg, warm compresses, ocular lubricants, artificial tears) and moving to office-based therapies (eg, thermal pulsation devices) and prescription medications as needed (7). Prescription treatment options for dry eye disease include the immunomodulator cyclosporin (formulated as a 0.05% ophthalmic emulsion (13), a 0.09% nanomicellar ophthalmic solution (14), and a 0.1% water-free solution (15, 16)), lifitegrast (a lymphocyte function–associated antigen-1 antagonist) ophthalmic solution (17–19), and varenicline (a cholinergic agonist) nasal spray (20). Loteprednol etabonate 0.25% ophthalmic suspension is a corticosteroid indicated for short-term treatment of dry eye flare-ups (21). However, none of these pharmacologic agents target the underlying cause of dry eye disease, namely excessive evaporation (7).

Perfluorohexyloctane ophthalmic solution (MIEBO^®^, previously NOV03) was recently approved by the US Food and Drug Administration for treatment of the signs and symptoms of dry eye disease (22). Because this new topical treatment consists solely of perfluorohexyloctane (a semifluorinated alkane), it is both water-free and preservative-free (23, 24). Perfluorohexyloctane has been shown to be present in tears through at least 6 hours in a rabbit pharmacokinetic study (Krösser S, et al. IOVS 2018;59:ARVO E-Abstract 2656) and forms a layer on the tear film surface to prevent evaporation of the underlying aqueous layer (22, 25). Thus, perfluorohexyloctane can act as a potential substitute for the dysfunctional tear film lipid layer in patients with Meibomian gland dysfunction (25).

Consistent results have been observed across clinical trials of perfluorohexyloctane in patients with dry eye disease with clinical signs of Meibomian gland dysfunction. In a phase 2 study (SEECASE), perfluorohexyloctane, administered either 2 or 4 times daily, demonstrated significantly greater improvement in signs and symptoms of dry eye disease compared with an isotonic saline (0.9% solution) control treatment (26). Similarly, 2 randomized phase 3 studies (GOBI (27) and MOJAVE (28)) found that perfluorohexyloctane significantly reduced dry eye disease signs (corneal fluorescein staining) and symptoms (eye dryness, eye burning/stinging) relative to a hypotonic saline (0.6% solution) control. In all clinical trials, perfluorohexyloctane was shown to have a favorable safety and tolerability profile (26–28).

The aim of this pooled analysis of data from GOBI and MOJAVE was to further evaluate treatment effects of perfluorohexyloctane in the overall population of patients with dry eye disease with clinical signs of Meibomian gland dysfunction and in patient subgroups defined by age, sex, and disease severity. Use of pooled data provides greater statistical power to perform subgroup analyses and increases the likelihood of identifying uncommon adverse events.

## Methods

### Study design and participants

Data were pooled from two phase 3 studies that evaluated the efficacy and safety of perfluorohexyloctane in patients with dry eye disease with clinical signs of Meibomian gland dysfunction: GOBI (NCT04139798) and MOJAVE (NCT04567329). This research was reviewed by an institutional review board and conforms with the principles and applicable guidelines for the protection of human subjects in biomedical research.

Methodology and results from each study have been described previously (27, 28). Homogeneity in study design and treatment duration allowed for pooling of patient-level data from GOBI and MOJAVE. Both were multicenter, randomized, double-masked, saline-controlled studies in which patients administered either perfluorohexyloctane ophthalmic drop or hypotonic saline (0.6% solution, preserved with benzalkonium chloride) 4 times daily for 8 weeks.

Study participants were adults (≥18 years) with a self-reported history of dry eye disease for 6 months who had ≥1 eye with tear film break-up time ≤5 seconds, ocular surface disease index score ≥25, Schirmer I test (without anesthesia) score ≥5 mm, total Meibomian gland dysfunction score ≥3 (0- to 15-point scale rating secretion from 5 central Meibomian glands on the lower eyelid; higher scores indicate greater dysfunction), and total corneal fluorescein staining score ≥4 and ≤11 according to the National Eye Institute scale (0- to 15-point scale; 0 to 3 points for each of 5 corneal areas: superior, inferior, central, nasal, temporal). If both eyes met inclusion criteria, the study eye was the eye with the higher (ie, worse) total corneal fluorescein staining score. All patients who were randomized and received study medication (perfluorohexyloctane or saline) in the GOBI or MOJAVE studies were included in this pooled analysis (pooled full analysis set).

### Study assessments and endpoints

Efficacy assessments included signs and symptoms of dry eye disease. Efficacy was assessed at Week 2 (Day 15 ± 1), Week 4 (Day 29 ± 2), and Week 8 (Day 57 ± 2). The primary sign endpoint was change from baseline at Week 8 in total corneal fluorescein staining score, and the primary symptom endpoint was change from baseline at Week 8 in visual analog scale eye dryness score (0 to 100). Key secondary endpoints were change from baseline in (1) visual analog scale eye dryness score at Week 2, (2) total corneal fluorescein staining score at Week 2, (3) visual analog scale burning/stinging score at Week 8, and (4) central corneal fluorescein staining score at Week 8.

Ocular and nonocular adverse events were monitored throughout the study. Other safety assessments included slit-lamp biomicroscopy, dilated fundoscopy, and intraocular pressure.

### Statistical analyses

The pooled full analysis set was used for all analyses. For the primary and key secondary endpoints, change from baseline was evaluated using analysis of covariance models with terms for baseline value, treatment group, and study; the least-squares mean treatment difference for perfluorohexyloctane versus saline was calculated. Efficacy on the primary endpoints was analyzed for patient subgroups by age (18 to <65 years and ≥65 years), sex (male and female), and baseline symptom severity: total corneal fluorescein staining score <7 and ≥7, visual analog scale eye dryness score <70 and ≥70, Meibomian gland dysfunction score <7 and ≥7, Schirmer I test <10 mm and ≥10 mm. Treatment differences for perfluorohexyloctane versus saline were summarized using forest plots (29). Logistic regression models, adjusting for baseline score, were used to analyze responder rates at Week 8 for the total corneal fluorescein staining score (defined as improvement of ≥3 points on the National Eye Institute scale) and eye dryness (defined as ≥30% reduction in the visual analog scale score). For each responder analysis, the odds ratio was calculated for perfluorohexyloctane versus saline control treatment.

## Results

### Patients

**Figure 1:**
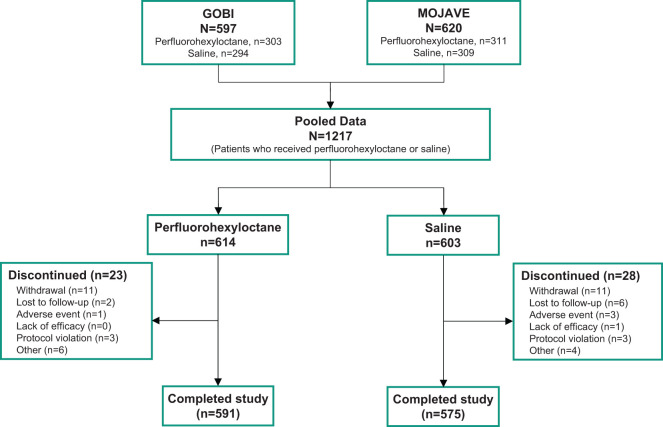
Patient disposition.

The pooled full analysis set included 1217 patients, 614 in the perfluorohexyloctane group and 603 in the saline control group. In all, 591 patients in the perfluorohexyloctane group (96.3%) and 575 patients in the saline control group (95.4%) completed the study (**Figure 1**). Demographic and baseline disease characteristics were balanced across treatment groups (**Table 1**). Most of the patients were female (75.7%), and more than one-third (39.2%) were at least 65 years of age.

**Table 1:** Demographic and baseline characteristics, pooled full analysis set.

Characteristic	Perfluorohexyloctane (n=614)	Saline (n=603)
Age, y, mean (min, max)	56.8 (19, 87)	57.6 (19, 88)
≥65 years, n (%)	235 (38.3)	242 (40.1)
Female, n (%)	469 (76.4)	452 (75.0)
Race, n (%)		
White	456 (74.3)	459 (76.1)
Black	76 (12.4)	75 (12.4)
Asian	70 (11.4)	55 (9.1)
Multiple/other	12 (2.0)	14 (2.3)
Baseline ocular characteristics		
Total corneal fluorescein staining score, study eye	6.9 (1.9)	6.9 (2.0)
Visual analog scale eye dryness score	65.6 (19.3)	65.5 (19.3)
Visual analog scale burning/stinging score	51.5 (26.3)	50.2 (26.4)
Meibomian gland dysfunction total score	7.6 (3.3)	7.9 (3.3)
Tear film breakup time, study eye, s	3.2 (0.9)	3.2 (0.9)
Schirmer I test (no anesthesia), study eye, mm	12.3 (7.9)	12.3 (7.8)
Ocular Surface Disease Index score	54.6 (17.5)	55.1 (17.1)
Best-corrected visual acuity, logMAR	0.07 (0.1)	0.08 (0.1)

Mean (standard deviation) unless stated.

logMAR, logarithm of the minimum angle of resolution.

### Efficacy

**Figure 2:**
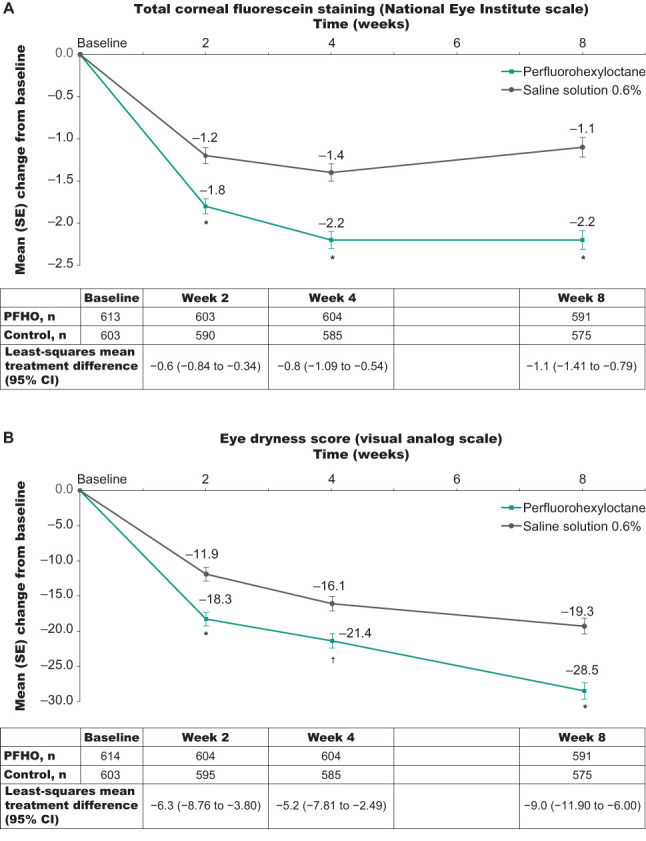
Change from baseline across the 8-week treatment period for the primary efficacy outcomes. **(A)** Total corneal fluorescein staining score (National Eye Institute scale). **(B)** Eye dryness score (visual analog scale). Analyses using pooled full analysis set. *p<0.0001 for perfluorohexyloctane versus saline control; ^†^p=0.002 for perfluorohexyloctane versus saline control. PFHO, perfluorohexyloctane.

**Figure 3:**
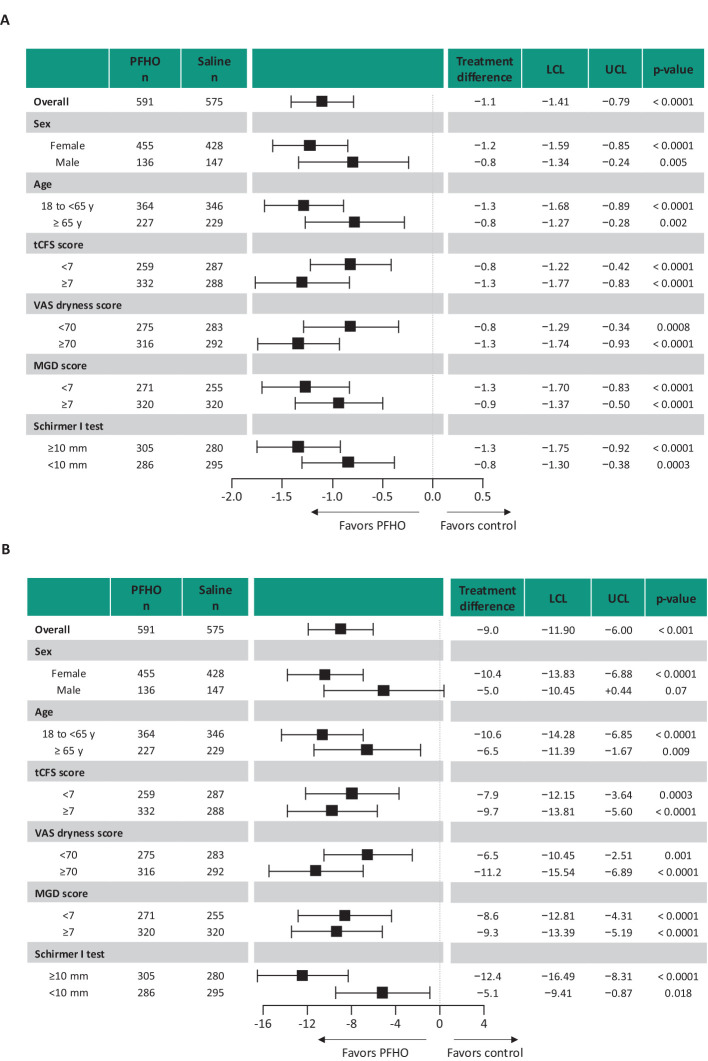
Change from baseline for the primary efficacy outcomes at Week 8 by patient subgroup. **(A)** Total corneal fluorescein staining score (National Eye Institute scale). **(B)** Eye dryness score (visual analog scale). Analyses using pooled full analysis set. Boxes represent the estimate of the difference between treatments (least-squares mean). Horizontal lines are 95% confidence limits. LCL, lower confidence limit; MGD, Meibomian gland dysfunction; PFHO, perfluorohexyloctane; tCFS, total corneal staining score; UCL, upper confidence limit; VAS, visual analog scale.

At all assessments, patients treated with perfluorohexyloctane showed significantly greater improvement in both total corneal fluorescein staining score and visual analog scale eye dryness score than patients in the saline control group (**Figures 2A, B**). For total corneal fluorescein staining score, least-squares mean change from baseline at Week 8 was −2.2 in the perfluorohexyloctane group and −1.1 in the saline control group; the least-squares mean treatment difference was −1.1 (95% CI: −1.41 to −0.79; p<0.0001). For the visual analog scale eye dryness score, least-squares mean change from baseline at Week 8 was −28.4 in the perfluorohexyloctane group and −19.4 in the saline control group; the least-squares mean treatment difference was −9.0 (95% CI: −11.90 to −6.00; p<0.0001). In the patient subgroup analyses, improvements in the total corneal fluorescein staining score were significantly greater for perfluorohexyloctane compared with the saline control in all subgroups evaluated: males and females, older (≥65 years) and younger patients, and those with more and less severe signs or symptoms of dry eye disease at baseline (**Figure 3A**). Improvements in the visual analog scale eye dryness score were significantly greater for perfluorohexyloctane versus saline in all patient subgroups evaluated, except for males, in which there was a numerical difference favoring perfluorohexyloctane that did not reach statistical significance (p=0.07; **Figure 3B**). Improvements in all key secondary endpoints, which included change from baseline at Week 8 for an additional sign (central corneal fluorescein staining) and symptom (burning/stinging) of dry eye disease, favored perfluorohexyloctane over saline control treatment (**Table 2**).

**Table 2:** Change from baseline key secondary endpoints, pooled full analysis set.

Endpoints	Perfluorohexyloctane (n=614) least-squares mean change	Saline (n=603) least-squares mean change	Least-squares mean treatment difference (95% CI)	p-value
Total corneal fluorescein staining score (study eye), Week 2	–1.78 (n=603)	–1.18 (n=590)	–0.59 (–0.84 to –0.34)	<0.0001
Visual analog scale eye dryness score, Week 2	–18.2 (n=604)	–11.9 (n=595)	–6.3 (–8.76 to –3.80)	<0.0001
Visual analog scale burning/stinging score, Week 8	–22.5 (n=590)	–16.1 (n=574)	–6.5 (–9.24 to –3.67)	<0.0001
Central corneal fluorescein staining score (study eye), Week 8	–0.41 (n=591)	–0.12 (n=575)	–0.29 (–0.37 to –0.20)	<0.0001

**Figure 4:**
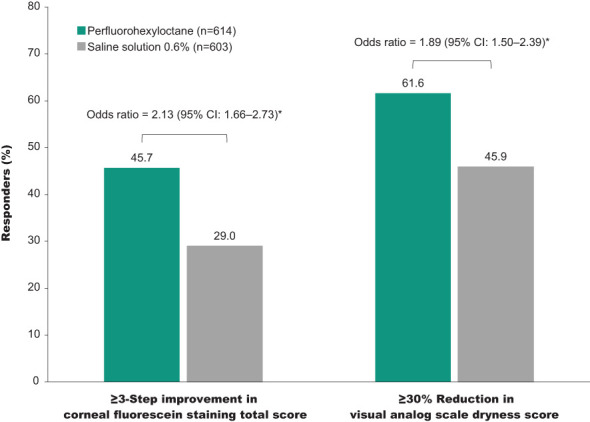
Responder rates at Week 8. Responder rates for total corneal fluorescein staining score and eye dryness score (visual analog scale), pooled full analysis set. *p<0.0001 for perfluorohexyloctane versus saline control.

Response rates were significantly greater for perfluorohexyloctane versus the saline control for all definitions of treatment response. At Week 8, the percentage of corneal fluorescein staining responders (≥3-step improvement in total corneal fluorescein staining score) was 45.7% in the perfluorohexyloctane group and 29.0% in the saline control group; the odds ratio for perfluorohexyloctane versus saline control was 2.1 (95% CI: 1.66–2.73; p<0.0001; **Figure 4**). The proportion of eye dryness responders (≥30% reduction in visual analog scale eye dryness score) at Week 8 was 61.6% in the perfluorohexyloctane group and 45.9% in the saline control group; the odds ratio for perfluorohexyloctane versus saline control was 1.9 (95% CI: 1.50–2.39; p<0.0001).

### Safety

The overall incidence of ocular adverse events was 11.2% in the perfluorohexyloctane group and 10.0% in the saline control group. Ocular adverse events considered by the investigator to be drug-related occurred in 6.4% of patients who received perfluorohexyloctane and 5.0% of patients who received the saline control (**Table 3**). The most common adverse event was blurred vision (incidence of 2.1% in the perfluorohexyloctane group and 0.3% in the saline control group), which was typically mild and transient. Ocular adverse events led to treatment discontinuation in 1 patient in the perfluorohexyloctane group (eye irritation) and 3 patients in the saline control group (conjunctivitis, dry eye, punctate keratitis). For other ocular safety assessments, no clinically meaningful changes were observed in either treatment group for best-corrected visual acuity, intraocular pressure, slit-lamp examinations, or dilated fundoscopy examinations.

**Table 3:** Summary of ocular adverse events.

Parameter	Perfluorohexyloctane (n=614) n (%)	Saline (n=603) n (%)
Patients with ≥1 ocular adverse event^a^	69 (11.2)	60 (10.0)
Mild	61 (9.9)	54 (9.0)
Moderate	7 (1.1)	5 (0.8)
Severe	1 (0.2)	1 (0.2)
Drug-related ocular adverse event^b^	39 (6.4)	30 (5.0)
Serious ocular adverse event	0 (0)	0 (0)
Ocular adverse event leading to discontinuation	1 (0.2)	3 (0.5)
Most common ocular adverse events^c^		
Vision blurred	13 (2.1)	2 (0.3)
Conjunctival hyperemia	5 (0.8)	6 (1.0)
Ocular hyperemia	6 (1.0)	1 (0.2)

a Patients instilled drops in both eyes; values represent n (%) of patients with an adverse event in either eye.

b Considered by the investigator as suspected/related to study medication.

c Incidence ≥1% in either treatment group.

The incidence of nonocular adverse events was 6.5% in patients receiving perfluorohexyloctane and 4.6% in those receiving saline. There were no nonocular adverse events leading to treatment discontinuation. One patient in the saline control group had a serious nonocular adverse event (chest pain) that was not considered drug-related.

## Discussion

This analysis of pooled data from the GOBI and MOJAVE studies demonstrated that perfluorohexyloctane was statistically superior to hypotonic saline for improving both the signs and symptoms of dry eye disease. Onset of effect was rapid, beginning as early as Week 2 (the first scheduled assessment), and sustained through the end of the 8-week treatment period. The results of this pooled analysis reflect the significant findings of the individual studies, which were notable for the consistent improvement observed with perfluorohexyloctane on multiple signs and symptoms of dry eye disease (27, 28).

The efficacy of perfluorohexyloctane was demonstrated across multiple patient subgroups based on age, sex, and baseline disease severity. Notably, the efficacy of perfluorohexyloctane for reducing the signs and symptoms of dry eye disease was evident regardless of whether patients had mild or moderate disease severity at baseline assessment. This is an important finding, as it points to the efficacy of perfluorohexyloctane for improving the symptoms of a wide range of patients suffering from evaporative dry eye. Perfluorohexyloctane was efficacious in both older and younger patients and in both males and females, although the difference between perfluorohexyloctane and saline did not reach statistical significance in one analysis (visual analog scale eye dryness score in males), possibly due to limited sample size. In each study, approximately 1 in 4 patients enrolled were male, consistent with known prevalence rates by sex (30).

The efficacy of perfluorohexyloctane was also evaluated in an independent, but similarly designed, multicenter study conducted in China (31). Treatment differences on study outcomes (eg, fluorescein staining, dry eye disease symptoms) for perfluorohexyloctane versus hypotonic (0.6%) saline were generally consistent with findings of this pooled analysis (**Table 4**), although superiority of perfluorohexyloctane was not demonstrated for central corneal fluorescein staining in the Chinese study.

**Table 4:** Comparison of outcomes with an independent study conducted in China.

Endpoints	Pooled analysis			Tian et al., 2023 (31)		
	PFHO (n=614)^a^	Placebo (n=603)^a^		PFHO (n=156)^a^		Placebo (n=156)^a^
tCFS score at Week 8						
LS mean change from baseline	–2.2	–1.1		–3.8		–2.7
LS mean treatment difference (95% CI)	–1.1 (–1.4 to –0.8) p<0.0001			–1.1 (–1.7 to –0.5) p<0.001		
VAS eye dryness score at Week 8						
LS mean change from baseline	–28.4	–19.4		–38.6		–28.3
LS mean treatment difference (95% CI)	–9.0 (–11.9 to –6.0) p<0.0001			–10.4 (–15.1 to –5.6) p<0.001		
VAS burning/stinging score at Week 8						
LS mean change from baseline	–22.5	–16.1		-26.7		-18.7
LS mean treatment difference (95% CI)	–6.5 (–9.2 to –3.7) p<0.0001			–7.9 (–13.1 to –2.8) p<0.01		
cCFS score at Week 8						
LS mean change from baseline	–0.4	–0.1		–0.6		–0.4
LS mean treatment difference (95% CI)	–0.3 (–0.4 to –0.2) p<0.0001			–0.2 (–0.4 to 0.0) p=0.12		

a N values vary slightly based on data availability.

cCFS, central corneal fluorescein staining; LS, least-squares; PFHO, perfluorohexyloctane; tCFS, total corneal staining score; VAS, visual analog score.

The safety and tolerability profile of perfluorohexyloctane was similar to that of hypotonic saline. In the pooled analysis population of 614 patients who received perfluorohexyloctane, only 1 patient discontinued treatment due to an adverse event, compared with 3 of 603 patients who received the saline control. The incidence of ocular adverse events considered treatment-related was also similar: 6.4% for perfluorohexyloctane and 5.0% for saline. The most common adverse event in the perfluorohexyloctane group was blurred vision, which was reported in 2.1% of patients.

Perfluorohexyloctane is the first prescription eye drop to be evaluated in pivotal clinical studies in a population of dry eye disease patients specifically selected to have clinical signs of Meibomian gland dysfunction, and it is known that such patients have alterations in the tear film lipid layer, leading to excessive evaporation (7). The unique physiochemical properties of perfluorohexyloctane enable it to form a monolayer at the air–liquid interface of the tear film to inhibit tear evaporation (22). The anti-evaporative effects of perfluorohexyloctane were demonstrated using an *in vitro* gravimetric assay that evaluated the evaporation rate of saline alone and after the addition of perfluorohexyloctane, meibum lipids (collected from a healthy volunteer), or common over-the-counter artificial tear products (25). The addition of a layer of perfluorohexyloctane (100 μL) reduced the evaporation rate of saline by approximately 80%, whereas adding the same volume of artificial tears had no effect. The reduction in evaporation was approximately 4 times greater with 1 drop (11 μL) of perfluorohexyloctane versus an ~125-nm layer of meibum lipids. Perfluorohexyloctane is distributed primarily on the ocular surface (the therapeutic target for the treatment of signs and symptoms of dry eye disease), as well as within Meibomian glands as a potential drug depot, as shown in a pharmacokinetic study of single and multiple topical instillation(s) in rabbits (Krösser S, et al. IOVS 2022;59:ARVO E-Abstract 2656). Perfluorohexyloctane was present in tears through at least 6 hours and in Meibomian glands through at least 24 hours; there was negligible perfluorohexyloctane in the posterior chamber, and systemic exposure was also very low.

One limitation of the studies included in this analysis was the relatively brief treatment duration (8 weeks). The KALAHARI study, a 52-week extension of GOBI, has provided information about long-term treatment with perfluorohexyloctane. Among 208 patients who continued into KALAHARI, the most common ocular adverse events during the extension study were vitreous detachment (1.9%), allergic conjunctivitis (1.4%), blurred vision (1.4%), and increased lacrimation (1.4%). Improvements in signs and symptoms of dry eye disease observed during treatment with perfluorohexyloctane in GOBI were maintained during an additional 52 weeks of treatment. In patients who switched from the saline control in GOBI to treatment with perfluorohexyloctane in KALAHARI, improvements in signs and symptoms of dry eye disease were observed by Week 4 (the earliest assessment time point in KALAHARI) and were maintained for the duration of the study.

Other limitations of the studies included in this analysis were the exclusion of patients with severe dry eye disease (total corneal fluorescein staining score >11) and use of hypotonic saline as the control treatment. Because perfluorohexyloctane is a single-component ophthalmic drop, there is no vehicle to serve as a control. On the other hand, as hypotonic solutions have been shown to be effective in the treatment of dry eye disease (32–34), use of hypotonic saline added rigor to GOBI and MOJAVE and could be perceived as a study strength. Finally, while hypotonic saline was preserved with benzalkonium chloride (0.01%), this was not considered to be a confounder, given its low concentration and the relatively short study duration.

## Conclusion

The combined safety and efficacy data from this pooled analysis demonstrate that perfluorohexyloctane is well tolerated and efficacious for improving both the signs and symptoms of dry eye disease associated with Meibomian gland dysfunction, including in patients who reported more severe eye dryness prior to treatment and patients with varying demographic characteristics.

## Data Availability

The original contributions presented in the analysis are included in the article. Further inquiries can be directed to the corresponding author.
